# The Need for a New Specialist Professional Research System of “Pure” Medical Science

**DOI:** 10.1371/journal.pmed.0020285

**Published:** 2005-08-30

**Authors:** Bruce G Charlton, Peter Andras

**Affiliations:** **1**University of Newcastle upon TyneNewcastle upon TyneUnited Kingdom

Awasthi et al.'s discussion of the future of academic medicine [1] is stimulating, but the primary focus of policy should be enhancing scientific progress in medicine. Science policy should address the decline in major, clinically relevant “breakthroughs” over recent decades [2].

Medical research has become mostly an “applied” science, which implicitly aims at steady progress by an accumulation of small improvements, each increment having a high probability of validity. Applied medical science is therefore a social system of communications for generating pre-publication peer-reviewed knowledge ready for implementation [3]. However, the need for predictability dictated by peer reviewing of research funding and the need for a high probability of validity in published research makes modern medical science risk-averse. This has led to a decline in major therapeutic breakthroughs where new treatments for new diseases are required [2].

There is a need for the evolution of a specialized professional research system of pure medical science, where the major evaluation of validity occurs (in the manner of classic sciences) post-publication and by peer usage, rather than peer review [3,4]. The role of pure medical science would be to generate and critically evaluate radically novel and potentially important theories, techniques, therapies, and technologies.

Pure science ideas typically have a lower probability of being valid, but have the possibility of much greater benefit if they turn out to be true [5]. The domination of medical research by “applied” criteria means that even good ideas from pure medical science are typically ignored or rejected as being too speculative. It is possible to publish radical and potentially important ideas in medical science, but at present there is no formal mechanism by which pure science publications may be received, critiqued, evaluated, and extended to become suitable for “application”.

Pure medical science needs to evolve to constitute a typical specialized scientific system of formal communications among a professional community with close research groupings, journals, meetings, and electronic and Web communications—like any other science. However, the pure medical science system would have its own separate aims, procedures for scientific evaluation, institutional organization, funding, and support arrangements, and it would have a separate higher professional career path with distinctive selection criteria. For instance, successful leaders of pure medical science institutions would need different qualities from many of the current leaders of medical science, and would need to be selected on the basis of their specialized cognitive aptitudes and their record of having generated science-transforming ideas.

The main “market” for pure medical science would be “applied” medical scientists who need radical strategies to solve important clinical problems that are not yielding to established methods. Pure medical science units might then arise as an elite grouping linked to existing world-class applied medical research institutions. The direct financial stimulus to create elite pure medical science institutions might come from the leadership of academic “entrepreneurs” and imaginative patrons in the major funding foundations.

## References

[pmed-0020285-b1] Awasthi S, Beardmore J, Clark J, Hadridge P, Madani H (2005). Five futures for academic medicine. PLoS Med.

[pmed-0020285-b2] Charlton BG, Andras P (2005). Medical research funding may have over-expanded and be due for collapse. QJM.

[pmed-0020285-b3] Charlton BG (2004). Conflicts of interest in medical science: Peer usage, peer review and ‘CoI consultancy’. Med Hypotheses.

[pmed-0020285-b4] Charlton BG, Andras P (2005). The future of ‘pure’ medical science: The need for a new specialist professional research system. Med Hypotheses.

[pmed-0020285-b5] Charlton BG (2004). Inaugural editorial. Med Hypotheses.

